# Electric vehicle adoption intentions among UK residents parking in shared and public spaces

**DOI:** 10.1007/s11116-024-10518-0

**Published:** 2024-07-29

**Authors:** Hannah Budnitz, Toon Meelen, Tim Schwanen

**Affiliations:** 1 Transport Studies Unit, University of Oxford, Oxford, UK; 2 Copernicus Institute, Faculty of Geosciences, Utrecht University, Utrecht, The Netherlands

**Keywords:** Electric vehicles, Adoption, Theory of planned behaviour, Residential parking, Environmental norms, United Kingdom

## Abstract

A lack of charging opportunities is cited as one of the main barriers to the adoption of plug-in battery electric vehicles (BEVs). Charging opportunities are even more limited for the millions of households across Europe without the space to charge a vehicle at home, but research has not previously targeted these households. This study aims to fill this gap by assessing whether and how such residential parking constraints shape norms, attitudes and perceived behavioural control over charging, and thus intentions to adopt a BEV. Data from a nationally representative survey of car drivers in the UK without a private driveway or garage captures the perspective of these individuals who have different characteristics from early adopters of BEVs. The results show that personal norms around the environmental benefits of BEVs and positive attitudes towards BEV technology have the greatest influence on intentions to adopt, whilst social norms and perceived behavioural control over charging are less influential. The results also show that high income men are most likely to hold positive social norms that boost their intentions to adopt BEVs, whilst geographical context affects both social norms and perceived behavioural control over charging. In summary, the psychological approach applied shows that a more inclusive transition to BEVs is best supported by policies that first focus on emphasising their environmental benefits and that they are fun and economical to drive, and then look to increase social expectations and awareness of BEVs and charging infrastructure.

**Supplementary Information:**

The online version contains supplementary material available at 10.1007/s11116-024-10518-0.

## Introduction

The electrification of private cars is part of a major socio-technical transition occurring in the transport sector as actors operating at multiple scales seek to respond to the environmental imperative to decarbonise (Geels 2012). Government policy support has helped plug-in, hybrid and full, battery-powered electric vehicles (EVs), which in transition terms are considered niche innovations, reach almost 10% of global car sales in 2021, suggesting momentum towards widespread adoption (Geels et al. 2017; IEA, 2022). Europe has the highest compound annual growth rate for EV sales over the last five years, and the share of electric car registrations in the United Kingdom (UK) in 2021 rose to almost 20% (IEA, 2022). However, the proportion of EVs in the national fleet in 2021 was less than 2% (less than 1% full battery-powered or BEVs), and the UK government Department for Transport (DfT) has identified “*world-class charging infrastructure*” as “*fundamental*” to decarbonising road transport and supporting widespread adoption of BEVs (DfT, 2022, p4). Academic literature and reviews of numerous empirical studies also evidence that a lack of charging infrastructure is a barrier to mass EV adoption globally (Biresselioglu et al. 2018; Hardman et al. 2018; Rezvani et al. 2015; Singh et al. 2020; Wicki et al. 2023). Yet there is less research into the norms, perceptions and attitudes, and intentions to adopt EVs, and particularly BEVs, for those most reliant on local, public charging infrastructure. This study aims to fill that gap.

The amount of public charging infrastructure is increasing rapidly in the UK and elsewhere. For example, when the survey in this study was conducted in spring 2020, there were less than 18,000 public charging points in the UK, whilst the most recent figures from April 2023 show more than 40,000 (DfT, 2023). Nonetheless, there is concern that publicly available charging facilities are not keeping pace with BEV sales nor meeting the 2014 European Union recommendations of one public charger per ten EVs and more recently 1 kW of publicly available charge per BEV in the large markets of France, Germany and the UK (Biresselioglu et al. 2018; IEA, 2022). Furthermore, with a lower proportion of single-family, detached dwellings with garages and driveways, these large European markets cannot rely as much on home charging as the USA or adoption frontrunner Norway (Funke et al. 2019; IEA 2022).

Thus, whilst early adopters of EV technology in North America and across Europe usually charge their vehicles privately at home, and the ability to charge at home is expected to continue to be important to adoption, that ability is not universal (Axsen et al. 2016; Bailey et al. 2015; Funke et al. 2019; Hardman et al. 2018; Patil et al. 2023; Vassileva and Campillo 2017). Azarova et al. (2020) estimate that over 40% of Europeans live in multi-dwelling buildings. In the UK, where parking on-street in front of dense, low-rise housing is common, the DfT (2022) estimates around 30% of households will be reliant on public charging, and that local provision will be required independent of charging infrastructure designed to support long-distance BEV driving. This study focuses on these households, who have not been targeted by previous research, which has been skewed towards ‘early adopters’ (Rogers 2003) and given limited attention to the impact of residential parking circumstances on BEV adoption intentions (Li et al. 2017; Singh et al. 2020; Wicki et al. 2023).

The lack of attention to parking circumstances in BEV adoption studies is problematic for at least two reasons. First, from a social justice perspective, it is important to acknowledge the differences in the abilities of different social groups to adopt BEVs, such as distinguishing between those who can or cannot charge from home (Sovacool et al. 2019a). Research targeting EV ‘early adopters’, whether of BEVs or hybrid vehicles, reflects the characteristics and motivations of that group, who are mostly wealthier men and homeowners with access to charging at home (Chakraborty et al. 2019; Corradi et al. 2023; Hardman et al. 2021; Vassileva and Campillo 2017; Wolbertus and Gerzon, 2018). An Irish study suggests that these wealthier early adopters are also the prime beneficiaries of grants to install home chargers (Caulfield et al. 2022). Furthermore, electricity for domestic charging in the UK has so far been taxed at only 5% compared to a rate of 20% at public charge points, and homeowners can enjoy the continued convenience of parking at home, with additionally avoiding journeys to recharge. These compound benefits do not accrue to other groups, so findings from studies using data from such groups cannot be seen as representative of the needs, norms, and attitudes of future user groups with different characteristics and parking circumstances, and thus cannot inform socially just policy interventions (Schwanen 2021).

Second, from a technological development and marketing perspective, many of these studies implicitly assume that the social and psychological motivations and barriers of early adopters of EVs are similar to those of the ‘early’ and ‘late majorities’ (Rogers 2003). Yet for the diffusion of other sustainability-oriented technologies, such as solar photovoltaics, economic factors were found to replace the primacy of environmental concern in later adoption decisions (Palm 2020). Such differences in motivation could have implications for information campaigns and infrastructure planning for future BEV use, beyond the instrumental need for more affordable BEVs or more equitable distribution of public charging infrastructure (Hardman et al. 2021; Patil et al. 2023). Thus, this study interrogates the norms and attitudes of those with different profiles to early adopters, and measures how their perceived ability to charge a BEV influences their intentions to adopt one.

An extended version of the Theory of Planned Behaviour (TPB) is employed, as it is one of the most widely used theoretical frameworks in the EV adoption literature (Singh et al. 2020). By considering the behavioural intention, rather than the behaviour itself, TPB enables a greater understanding of consumer acceptance and future purchase at a time when actual purchase of BEVs is still a minority decision (Ajzen 1991; Alzahrani et al. 2019; Egbue and Long 2012; Schuitema et al. 2013), especially among those without somewhere at home to charge one. In the next section, we explain the conceptual basis of this study and discuss how our work extends the TPB to account for not only the influence of residential parking circumstances, but also any heterogeneity within this group who may be reliant on public charging.

## Conceptual framework

### Adoption of electric vehicles

Review papers systematically assess empirical investigations into factors that influence EV^1^ adoption by individuals and households around the world, as well as the theoretical and socio-psychological models that underlie them (Corradi et al. 2023; Li et al. 2017; Liao et al. 2017; Singh et al. 2020; Wicki et al. 2023). Two common theoretical approaches reviewed are the Theory of Planned Behaviour (TPB) and the Norm Activation Theory or Model (NAM), which some studies combine by either using a staged approach or extending the psychological factors in TPB to include environmental values, environmental concern and/or personal norms (Haustein and Jensen 2018; Huijts et al. 2012; Klöckner 2014; Nayum et al. 2016; Wang et al. 2016). Other papers take a less psychology-oriented approach and consider the attributes of available EVs, such as cost and range, and the public charging infrastructure or financial incentives offered in a geographic area (Hidrue et al. 2011; Jensen et al. 2014; Kim et al. 2020). Socio-demographic characteristics, such as gender, age, vehicle ownership, and household income are often included, at least in descriptive statistics. However, out of hundreds of studies into the psychological, attitudinal effects on EV adoption, considerations of the impact of diverse residential parking circumstances within a population appear largely absent (Corradi et al. 2023; Liao et al. 2017; Singh et al. 2020). As parking and charging at home is so dominant among early adopters’ use of BEVs, this study focuses on measuring the psychological factors that influence BEV adoption among a subset of the population with alternative residential parking situations.

### The socio-psychological framework of EV adoption

The TPB is the most commonly used socio-psychological framework to understand individual intentions to switch from a conventional, fossil-fuel powered vehicle to an EV (Singh et al. 2020). In its most basic form, the framework considers the effects of three psychological constructs on an individual’s intentions to behave in a certain way (Ajzen, 1991):

- *Attitudes* pertaining to the behaviour;
- The *social norms* (expectations) for behaving that way, sometimes also called ‘subjective norms’; and.
- The perceived ability, opportunity and resources to behave that way, known as *Perceived Behavioural Control* (*PBC*).

Purchasing (or leasing) a new vehicle is an infrequent, potentially high-cost choice, which, when examined through the TPB, incorporates the understanding that psychological motivations are part of a decision-making process influenced by more than environmental norms (Klöckner 2014; Steg 2009). However, as EV adoption may be studied as a potentially environmentally-friendly decision, the TPB is often extended to enable the inclusion of moral aspects of taking such actions, often by including *personal norms* as described by the NAM (Alzahrani et al. 2019; Haustein and Jensen 2018; Klöckner and Blöbaum 2010; Nayum et al. 2016; Wang et al. 2016). Furthermore, previous studies of environmentally-motivated behaviour change indicate that the *personal norms* described by NAM may have more explanatory potential alongside the TPB as a measure of how they influence intentions rather than as an independent influence on behaviour (Abrahamse et al. 2009; Bamberg et al. 2007).

Finally, the environmental psychology literature has demonstrated the theoretical and empirical relevance of values or value orientations in influencing environmental behaviour. A distinction is made between three value orientations: egoistic, altruistic and biospheric (de Groot and Steg, 2010). People with the latter orientation hold the perceived impacts on the environment and ecosystems as guiding principles in their decision-making. Given that transport electrification is often framed as environmentally-motivated transition (Rezvani et al. 2015), the biospheric value orientation is included in the study.

### Defining TPB and NAM variables

In numerous studies, attitudes are shown to significantly influence intentions to adopt EVs, but there is no consistent approach to what constitutes measurements of attitudes in this context (Singh et al. 2020). Some studies frame attitudinal constructs in the TPB using affective opposites, such as ‘favourable’ and ‘unfavourable’, ‘pleasant’ and ‘unpleasant’ (Alzahrani et al. 2019; Nayum et al. 2016; Wang et al. 2016). Other studies, which use the TPB more implicitly, analyse how perceptions of the benefits of EV adoption influence the decision-making process, with a focus on attitudes as an exercise in weighing the costs and benefits of a major purchase (Axsen et al. 2016; Egbue and Lond, 2012; Schuitema et al. 2013; Sovacool et al. 2019b). These studies use statements expressing commonly assumed advantages and disadvantages of EV technology, such as purchase versus use costs, although they may also include affective variables, such as the appeal of innovative progress in batteries and EV technology. As our focus is on the influence of attitudes towards using rather than purchasing a BEV, we chose to use statements about the cost and affective attributes of BEV use for our attitudinal constructs in TPB, similar to Haustein and Jensen (2018).

Many studies have shown that social norms are influential within both the TPB and other frameworks, although some have found them less important than other psychological constructs such as attitudes and personal norms (Li et al. 2017; Rezvani et al. 2015; Singh et al. 2020). Wang et al. (2016), who used the TPB to study hybrid EV adoption intentions in China, noted that whilst social norms had the greatest effect of any construct in their study, this may be due to China’s social culture, in comparison to the individualism that heightens the effect of personal norms in European and North American studies. Conversely, White et al. (2022) find that social norms are consistently the most statistically significant mediating factor between the density of charging infrastructure and adoption intentions across three metropolitan areas in the United States. They conclude that public charging infrastructure access and visibility may be at least as important as actual quantity in fostering social norms and increasing adoption intentions (ibid.). Furthermore, research into residential parking in dense neighbourhoods in Poland and Germany suggests that parking on-street involves more social interaction and coordination of parking routines to maintain satisfaction in the local area despite parking pressures (Kurnicki 2020; Scheiner et al., 2020). These links between social norms and conducting parking or charging activities in public spaces suggests that social norms may be more influential among this study’s target group than among early adopters who can park and charge at home.

Meanwhile, personal norms that drive pro-environmental behaviour are influenced by values (Bockarjova and Steg, 2014; Nayum et al. 2016; Schuitema et al. 2013). Some studies unpick the question of whether adopting EVs is perceived by study participants as effective pro-environmental behaviour, considering that there is debate over whether all aspects of EV technology are environmentally friendly or have positive environmental impacts (Rezvani et al. 2015). This is not a debate that we engage with in our study, so statements of personal norms assume that BEV adoption is an environmentally-motivated behaviour. However, we did want to capture the influence of values on personal norms. Therefore, we use the empirically validated measurements of biospheric values by de Groot and Steg (2010), which include statements about ‘preventing pollution’ and ‘protecting the environment.’

Finally, Perceived Behavioural Control (PBC) is particularly relevant for this study, as it allows us to capture the perceived control over charging a BEV once purchased among a sample that may not have the most obvious form of control, namely the ability to install private, domestic charging. A lack of access to charging is one of the major barriers to EV adoption highlighted in the literature (Biresselioglu et al. 2018; White et al. 2022), but measures of PBC have not previously focused on charging opportunities. A Norwegian study of conventional car drivers found PBC to be the strongest predictor of intentions, but measured it as the perceived ability to purchase or afford a BEV (Simsekoglu and Nayum 2019). Other studies highlight range anxiety, defined in various ways, as a psychological barrier to adoption (Corradi et al. 2023; Haustein and Jensen 2018; White et al. 2022). One measure of range anxiety is included in our PBC construct, but our other measures of PBC focus on charging infrastructure and the *perceived* opportunities and ability to charge a BEV near the participant’s home.

### Personal characteristics

Various studies of EV adoption include sociodemographic variables, such as gender, education, income and age, often as control variables or to determine the representativeness of the study (Bamberg et al. 2007; Corradi et al. 2023; Li et al. 2017; Rezvani et al. 2015; Singh et al. 2020; Wang et al. 2016; Wicki et al. 2023). There are also studies indicating that values and particularly environmental values and motivations vary by gender (Bamberg et al. 2007; Stern et al. 1993). Surveys show that current EV owners and drivers are predominantly men, but that women participants have stronger environmental preferences and occasionally expressed greater interest in EV adoption (Chakraborty et al. 2019; Egbue and Long 2012; Philipsen et al. 2018; Simsekoglu and Nayum 2019; Sovacool et al. 2019c; Vassileva and Campillo 2017). Thus, the different observed effects of gender on BEV adoption make it an important variable to include (Wicki et al. 2023).

It is also important to consider the interaction between gender and income, in part because several studies of EV adoption from the Nordic region find that their sample is skewed not just towards men, but towards wealthier men (Chen et al. 2020; Haustein and Jensen 2018; Sovacool et al. 2019a, b; Vassileva and Campillo 2017). Haustein and Jensen (2018) raise the question as to whether the association of high status with BEV ownership is more common among men, influencing their subjective norms. Existing literature, however, does not analyse the differences in intentions between higher and lower income women or higher and lower income men. Income on its own is often included, as it clearly affects an individual’s or household’s ability to purchase an EV, and purchase cost remains an oft-cited and statistically significant barrier to adoption intentions (Simsekoglu and Nayum 2019; Wicki et al. 2022). Income also influences how attributes of public parking and charging options are valued (Azarova et al. 2020; Taylor, 2020). Thus, income is included, as is the interaction between gender and income, for these variables are relevant to both a household’s valuation of residential parking and charging and to BEV adoption.

### Situational variables related to residential parking

Finally, the literature on the provision of charging infrastructure required to support the spread of EVs highlights why existing residential parking is important (Anderson et al. 2018; Chakraborty et al. 2019; Funke et al. 2019; Patil et al. 2023). The charging behaviour of those who already drive an EV is closely aligned with parking behaviours and preferences, and studies suggest that this should guide policy for public parking and charging infrastructure and management (Wolbertus et al. 2018a, b). A study comparing recharging to refuelling concluded that BEV charging behaviour is shaped more by preferences for habitual locations and integration into daily routines than the refuelling of conventional vehicles (Philipsen et al. 2018). No location is more fundamental to daily routines than the home, as reflected in the importance of charging access at home to early adopters (Axsen et al. 2016; Hardman et al. 2018). Thus, Funke et al. (2019) aim to quantify the level of public charging required in different countries by comparing statistics on population density and the share of detached homes to better estimate the proportion of households who will *not* be able to charge at home. Conversely, we targeted our survey at those households.

Having a place at home to charge is not the sole consideration in how residential parking and public charging availability may influence BEV adoption. Various studies highlight the importance of spatial context and built environment to public charging awareness and availability. Some analyse the influence of variables like population density or settlement size on EV adoption, albeit with mixed results (Abotalebi et al. 2020; Brückmann et al. 2021; Haidar and Rojas 2022; Priessner et al. 2018). Guerra and Daziano (2020) describe that among their survey participants, those in dense, central residential areas in Philadelphia, USA were so willing to pay to reduce parking pressure and search time that they claimed to be interested in purchasing an EV if it meant the allocation of a dedicated parking and charging space. Studies on residential parking indicate that drivers prefer to park on-street in areas where on-street parking is both free and unrestricted, as this is seen as more convenient than available off-street space (Scheiner et al., 2020; Taylor, 2020). Wolbertus et al. (2018a) have found that proposed policies of providing more charging points on-street alongside residential parking spaces reserved exclusively for EVs in Dutch cities affect intentions to adopt BEVs. Sommer and Vance (2021) conclude that installing charging infrastructure in more densely populated locations in their German study encourages greater EV adoption per charge point than in less dense areas, creating a virtuous circle, and one attractive for commercial investment in further public charging in high density neighbourhoods. Finally, a spatial analysis in Ireland found that early adopters of BEVs are clustered around the cities with the highest population densities (Mukherjee and Ryan 2020).

In this way, the literature suggests that not only is a lack of private parking at home a barrier to EV adoption (Bailey et al. 2015; Hardman et al. 2018), but also that living in a densely populated area with limited parking space can affect how that barrier is perceived and how the installation of public charging is received. Thus, situational variables such as household density (number of households per kilometre squared), and whether or how residential parking is managed are included in our study. At the time of our survey, there was limited public on-street charging infrastructure in the UK, but Greater London was home to a third of the public charge points available in England, and had more than double the number of charge points per 100,000 population than the UK average (DfT 2023). This is why a Greater London dummy is also included in our model.

### Summary of variables

**Fig. 1:**
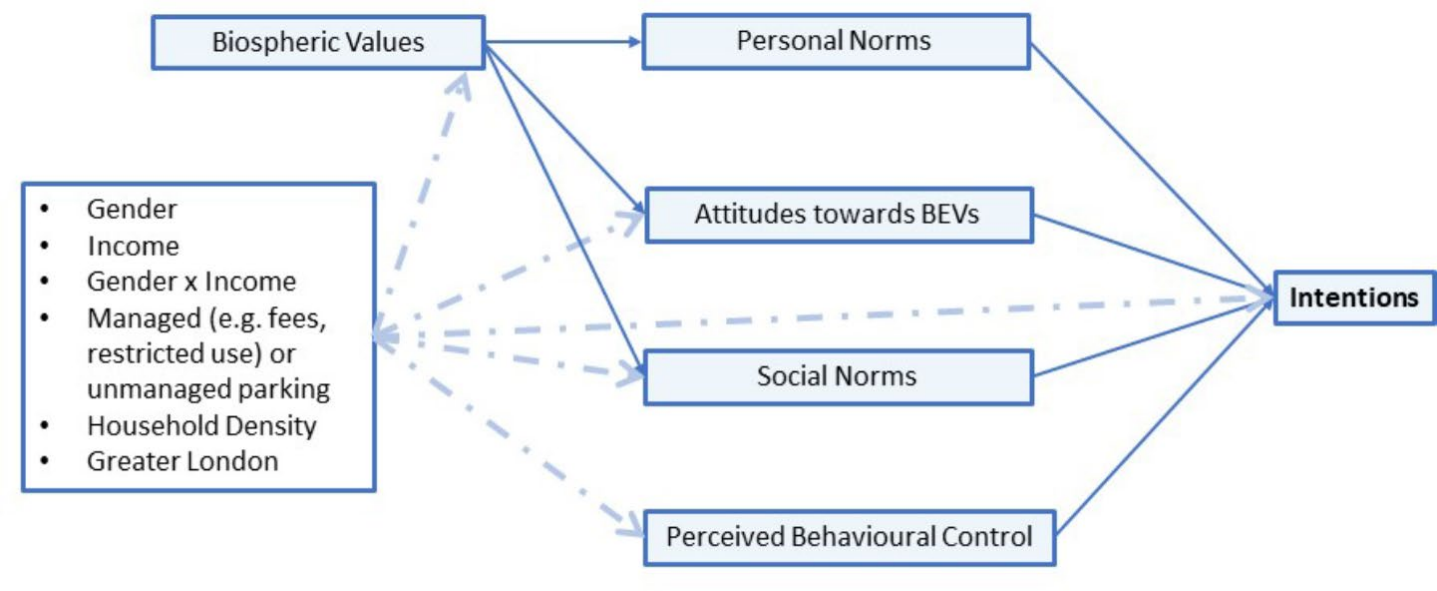
Conceptual framework of influences on intentions to adopt a BEV, with relationships between latent constructs indicated by solid arrows and the potential relationship of sociodemographic and geographic variables indicated by dotted arrows

In summary, our analysis presents a socio-psychological framework for estimating intentions to adopt a BEV that explicitly interrogates the norms, attitudes and PBC held by those without a private driveway on which to charge a BEV at home. Synthesizing from previous literature on parking, charging, EV adoption and other environmentally-motivated behaviours, we include biospheric values, personal norms, social norms, attitudes toward BEVs, PBC, gender, income, the interaction of gender and income, and situational variables related to residential parking management and public charging provision within the framework shown in Fig. 1 below.

The literature supports a modelling structure where biospheric values or environmental beliefs have an indirect effect on the intention to obtain a BEV, via attitudes and both personal and social norms; whilst the other four constructs have direct effects on intention (Abrahamse et al. 2009; Ajzen, 1991; Klöckner and Blöbaum, 2010; Simsekoglu and Nayum 2019). The model is then expanded to include not only gender and income, but also the interaction between them, and three geographic variables in order to situate the targeted participants’ spatial and social context within the wider conceptualisation of a population of car drivers who are unlikely to be able to charge at home.

## Data and measures

### Surveying those unlikely to be able to charge at home

In order to understand what influences intentions to adopt BEVs among those who are unlikely to be able to charge at home on their driveway, we hired Accent Marketing and Research Limited to run a UK-wide online panel survey, which was conducted in May-June 2020. The complete text presented to participants in the survey can be found in the Supplementary Information accompanying the online article. Quotas were set for gender and age and region to make the survey representative of the car-owning / driving population according to the English National Travel Survey (NTS), and then estimated for the other UK nations. The survey targeted those who would be unlikely to be able to recharge a vehicle easily and safely from their domestic electricity. In the UK, this is most commonly households who park on-street, but it was recognised that there are off-street residential parking areas that are not adjacent to the dwelling, may not have electricity available, and may be shared with neighbours. Therefore, a scoping question asked participants: *Do you have a driveway or attached garage at your home that fits ALL of the vehicles regularly driven by you or members of your household?* (see also Supplementary Information). Scoping questions were also included to filter out the quarter of UK households without access to a private vehicle and individuals who do not drive. The questions, format and ease of completion were tested in a pilot of the survey on 100 participants. Once quotas were reached for the final survey, a total of *N* = 2,001 questionnaires, excluding any identified as incomplete or falsely completed, were available for analysis.

Two individuals put their gender as ‘neither’ or ‘other’, and were excluded from consideration as this group was too small to consider separately in the analysis. Over 6% of participants did not respond to the income question and were also excluded, resulting in a sample size of 1,858 responses. At least partial postcodes were required to calculate the average density of households in the postcode districts where survey participants live, and missing, incomplete or unverifiable data resulted in a further reduction in the sample size to 1,774 responses.^2^ A further 24% of the participants indicated that, although they do not have sufficient private space for all the vehicles in their household, they usually park in their own parking garage or driveway. As this final quarter may be able to charge a BEV from their domestic electricity, they have also been excluded from this analysis. Finally, 69 participants, who indicated no desire to purchase a future vehicle, whether electric or not, were excluded, which leaves *N* = 1,281 participants for the analysis. As Table 1 shows, both the total sample and the final sample are skewed ever so slightly to younger age groups than England’s car-driving population. However, the final sample seems to be more representative of the wider UK population in terms of household income than the total sample, with 35% instead of 37% on household incomes over £40,000.

**Table 1:** Representativeness of survey sample and final sample for key socio-demographic variables

		Original Sample (*N* = 2,001)	Final Sample (*N* = 1,281)	Car drivers or car owning households, England^a^	Population GB^b^
Gender	Men	53%	54%	54%	49%
	Women	47%	46%	46%	51%
Age	Under 50	55%	56%	52.6%	54%
	Over 50	45%	44%	47.4%	46%
Household income ^c^	Up to £40,000	57%	65%	-	-
	Over £40,000	37%	35%	-	-
	Average (mean)			-	£34,200

^a^ National Travel Survey (NTS), DfT, 2018

^b^ Office for National Statistics (ONS), 2019 except mean income, estimated for the financial year ending 2018

^c^ Approximately 6% of this sample did not respond to the income question

Table 2 summarises the detailed responses to the situational variables on residents’ parking. Of the 1,281 participants, 49% said they usually park on street where there are no restrictions on when, where or who can park alongside the kerb. A further 20% park on streets where there are restrictions, which could include limits on time of day or duration of parking, or could restrict who is permitted to park in marked bays, for instance, residents’ only. Of the participants who parked on-street with restrictions, over half paid for parking, for example by means of a monthly or annual residents’ parking permit. Car parks or shared parking areas were identified by 31% of the sample as the location where they usually park when at home. About half of these were allocated to a specific individual or household (15% vs. 16% of respondents with unallocated spaces).

**Table 2:** Parking circumstances of survey participants

	On street, no restrictions	On street with restrictions	Personal space in a car park	Car park / parking area (unallocated)
Usual parking space when at home (number of participants)	631	255	189	206
Usual parking space when at home (% of total participants)	49%	20%	15%	16%
Pay for residential parking (% of participants using that type of parking space who pay)	1%	52%	17%	13%

**Fig. 2:**
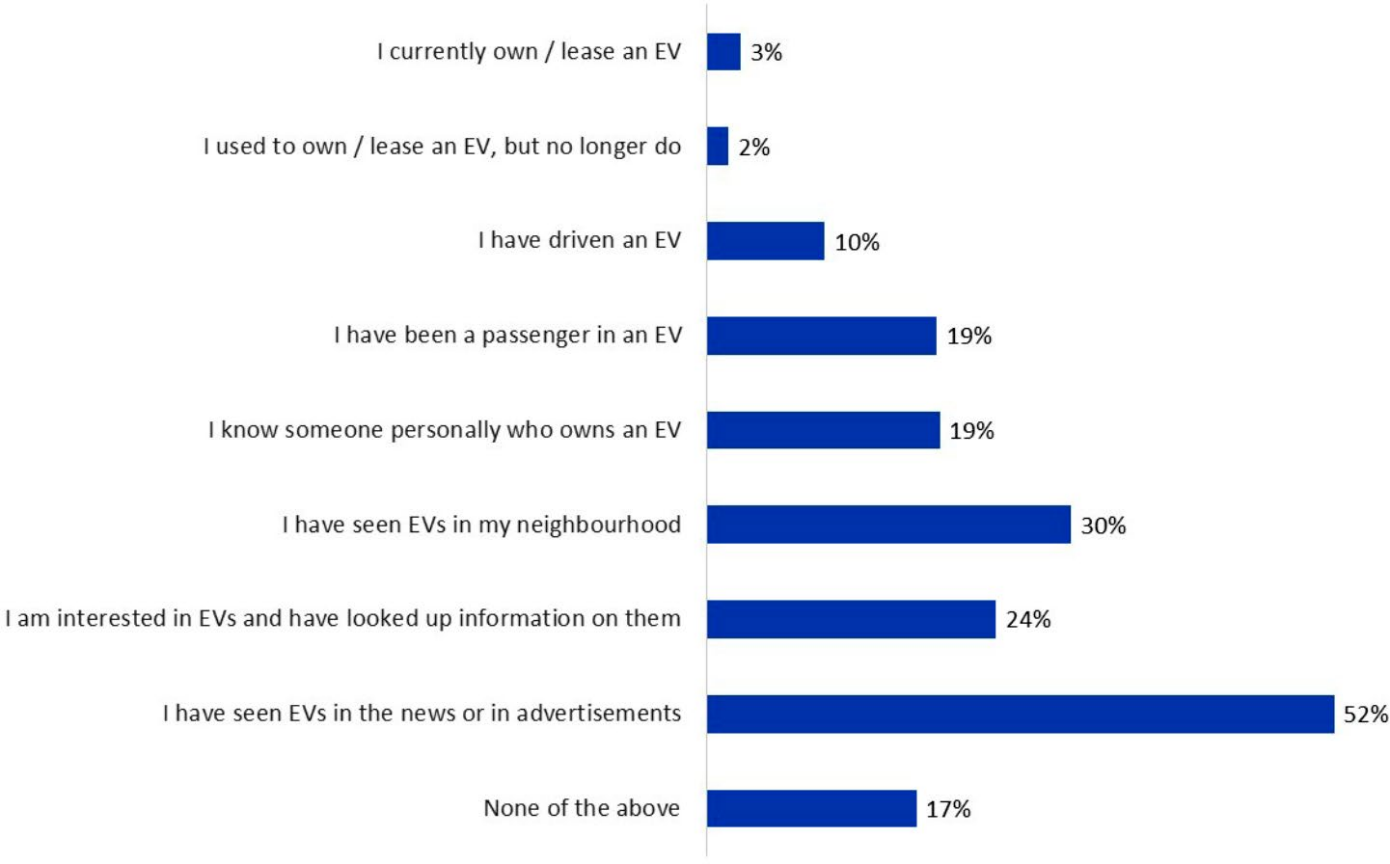
Percentage of 1,281 participants with different types of experience or awareness of BEVs; participants were allowed to tick multiple statements

Overall, the final sample neither reflects where the vast majority of current early adopters of EVs park and charge, nor exhibits the skew towards higher income men characterising some earlier studies. Few have much personal experience with BEVs as defined in the survey prior to the relevant section of questions.^3^ Some even lack a basic awareness as shown in Fig. 2.

### Measuring socio-psychological constructs

Participants were randomly presented with the fourteen statements shown in Table 3 to measure their norms, attitudes, and perceptions (PBC) towards BEVs. These, along with separate questions on intentions and biospheric values, were measured on five-point Likert scales, with sliding scale buttons as shown in the survey text included as Supplementary Information. To increase the validity of our constructs, our survey replicates the text of scale questions for personal and social norms, values, and intentions from previous studies where possible (Bamberg et al. 2007; Haustein and Jensen 2018; Klöckner and Blöbaum, 2010; Nayum et al. 2016; Wang et al. 2016; White et al. 2022). The two-item biospheric value scale (Groot and Steg, 2010) was included in the final section of the questionnaire. As mentioned in Sect. 2.3, our survey used statements reflecting common attitudes specific to owning and driving a BEV. Some of the measures in Schuitema et al. (2013) proved useful in phrasing these attitudes, as their study also tests the influence of socio-psychological constructs on EV adoption (although the set of constructs they considered differed from ours).

**Table 3:** Reliability of latent psychological constructs

	Mean	SD	Cronbach’s α
**Personal Norm**	3.03	1.03	0.89
I would feel a strong personal obligation to buy / lease an EV for my next vehicle to reduce carbon emissions and improve air quality.	3.10	1.14	
I would feel guilty if I did not purchase an EV for my next vehicle to reduce carbon emissions and improve air quality.	2.76	1.15	
Because of my own principles, I feel I should choose an EV for my next vehicle to reduce carbon emissions and improve air quality.	3.23	1.13	
**Attitudes towards BEVs**	3.41	0.67	0.76
EVs are a good option because they are cheap to run and maintain.	3.39	0.91	
EVs are an exciting new technology.	3.82	0.92	
Over the long term, an EV is a cheaper option than a diesel or petrol vehicle.	3.43	0.93	
EVs are more comfortable to drive than a conventional vehicle.	3.01	0.73	
**Social Norm**	2.60	0.86	0.76
Many of the people who are important to me (friends, family) own fuel efficient and environmentally friendly vehicles.	2.59	1.05	
People whose opinions I value would prefer that I adopt an EV when adopting a vehicle in the near future.	2.80	1.03	
People who are important to me expect me to choose an EV for my next vehicle.	2.40	1.06	
**Perceived Behavioural Control**	3.89	0.85	0.77
When driving an EV, I would always be worried about running out of charge.	3.93	0.94	
I think it would be difficult to park and charge an EV near my home if my household bought / leased one.	3.96	1.08	
I wouldn’t know how to charge an EV near my home.	3.75	1.15	
In my neighbourhood, I don`t know where I could charge an EV.	3.93	1.18	
**Intention to adopt a BEV**	3.03	1.04	0.93
I plan to replace my current vehicle with an EV.	2.85	1.08	
I will seriously consider leasing or buying an EV when I need a new vehicle.	3.23	1.17	
For my next vehicle, I want to buy / lease an EV.	3.01	1.10	
**Values biospheric**	4.05	0.85	0.90
Preventing pollution: protecting natural resources	3.99	0.91	
Protecting the environment: preserving nature	4.10	0.87	

Table 3 lists the statements that make up each socio-psychological construct. It provides the mean and standard deviation of responses on a scale of one, strongly disagree, to five, strongly agree across the sample of 1,281 participants. For the biospheric values, participants were asked to indicate how important each was as a guiding principle in their lives, on a scale from one, not important at all, to five, very important. Table 3 also shows that when using Cronbach’s alpha to test for consistency in the open statistical computing language and environment R (R Core Team, 2022), all constructs are above the threshold of 0.70 and can be considered robust.

Participants hold strong biospheric values and have broadly positive attitudes towards BEVs and personal norms are also fairly strong. The means for social norms are relatively low, reinforcing the insight from Fig. 2, that a low proportion of participants have friends, family or neighbours that already drive BEVs and can set an example. The average scores for PBC in Table 3 are high, reflecting the expected lack of confidence among participants in knowing when, where and how to charge a BEV. The above responses are used in a Structural Equation Model (SEM) to estimate their influence on participants’ intentions to switch. The scores for PBC are reversed for use in the model, as they were phrased negatively.

### Modelling choices

Structural Equation Modelling (SEM) is a general framework for modelling, in which equations are estimated as a system and variables can be included as dependent in one equation and as independent in others. A distinction is made between the structural model in which latent variables are related to each other, and a measurement model in which latent variables are constructed out of several observed variables. The analysis began with estimating the coefficients in the measurement model for the latent psychological constructs (Table 3), and in the structural model laying out the hypothesized relationships among the six latent psychological constructs of intentions, attitudes, social norms, PBC, personal norms and biospheric values (Fig. 1). In addition, associations among attitudes, social norms, PBC and personal norms were considered as some of the covariances between these constructs were markedly greater than zero. It was decided to leave the strongest of those relationships as covariance terms and not specify them as one-directional effects of one variable on the other, because no meaningful direction of influence could be established for any pair of the involved psychological constructs (see also Ajzen 2020).

Subsequently, the effects of various sociodemographic and geographic variables on the six psychological constructs were considered. Since these consist of only one observed variable (e.g. income), they are directly included in the structural model. Single equation OLS regression model specifications were estimated with the individual-specific average across the items contributing to a given psychological construct (Table 3) as dependent variables. These models were used to explore the strength of the relationships of sociodemographic and geographic characteristics with the six psychological constructs. Then the sociodemographic and geographic variables that had given statistically significant results – gender, income, the ‘gender x income’ interaction term, managed parking, household density, and a dummy for living in Greater London – were introduced into the SEM model. They were initially linked to the psychological constructs on which they had significant effects in the single equation models, but the possibility of significant and meaningful effects on other constructs was examined as well. Final model specifications were decided through an iterative process and based on a combination of three criteria – plausibility of interpretation, model goodness of fit, and parsimony of model specification.

Based upon our conceptual model detailed in Sect. 2.6, we used the lavaan package in R (Rosseel 2012) to apply the SEM methodology. The lavaan package uses maximum likelihood as the default method of estimation, which was deemed appropriate for our purposes considering the large sample size, endogenous and continuous dependent variable (ibid.). This methodology also follows the literature which uses SEM and maximum likelihood estimation to help describe the directional relationships between values, norms, attitudes and their effects on intended adoption in extended TPB frameworks (Bamberg et al. 2007; Wang et al. 2016).

## Results

### Measurement model results

Although the Cronbach’s alpha is informative in terms of the reliability of the latent constructs, it tells us little about the factorial structure of the measures or the loading of each statement within each construct. Therefore, we ran the measurement model, the outputs of which are shown in Table 4. This presents both the raw coefficients and standardised factor loadings. All individual coefficients are significant to *p* < 0.001 and the loadings further suggest internal consistency within constructs.

**Table 4:** Measurement model of latent constructs

	Coefficients	Standardised
**Values biospheric**		
Preventing pollution: protecting natural resources	1.000	0.924
Protecting the environment: preserving nature	0.910	0.882
**Personal Norm**		
I would feel a strong personal obligation to buy / lease an EV for my next vehicle to reduce carbon emissions and improve air quality.	1.000	0.884
I would feel guilty if I did not purchase an EV for my next vehicle to reduce carbon emissions and improve air quality.	0.914	0.801
Because of my own principles, I feel I should choose an EV for my next vehicle to reduce carbon emissions and improve air quality.	0.987	0.878
**Attitudes towards BEVs**		
EVs are a good option because they are cheap to run and maintain.	1.000	0.712
EVs are an exciting new technology.	1.033	0.726
Over the long term, an EV is a cheaper option than a diesel or petrol vehicle.	1.043	0.728
EVs are more comfortable to drive than a conventional vehicle.	0.565	0.504
**Social Norm**		
Many of the people who are important to me (friends, family) own fuel efficient and environmentally friendly vehicles.	1.000	0.505
People whose opinions I value would prefer that I adopt an EV when adopting a vehicle in the near future.	1.604	0.830
People who are important to me expect me to choose an EV for my next vehicle.	1.676	0.839
**Perceived Behavioural Control (all reversed)**		
When driving an EV, I would always be worried about running out of charge.	1.000	0.445
I think it would be difficult to park and charge an EV near my home if my household bought / leased one.	1.829	0.711
I wouldn’t know how to charge an EV near my home.	2.084	0.760
In my neighbourhood, I don’t know where I could charge an EV.	2.307	0.818
**Intention to adopt a BEV**		
I plan to replace my current vehicle with an EV.	1.000	0.897
I will seriously consider leasing or buying an EV when I need a new vehicle.	1.063	0.880
For my next vehicle, I want to buy / lease an EV.	1.042	0.921

The measurement model also showed an acceptable fit with the data, with a root mean square error of approximation (RMSEA) of 0.051 with confidence intervals of 0.047–0.055, a standardised root mean square residual (SRMR) of 0.041, and a Tucker Lewis Index (TLI) of 0.959.

### Structural model results

**Fig. 3:**
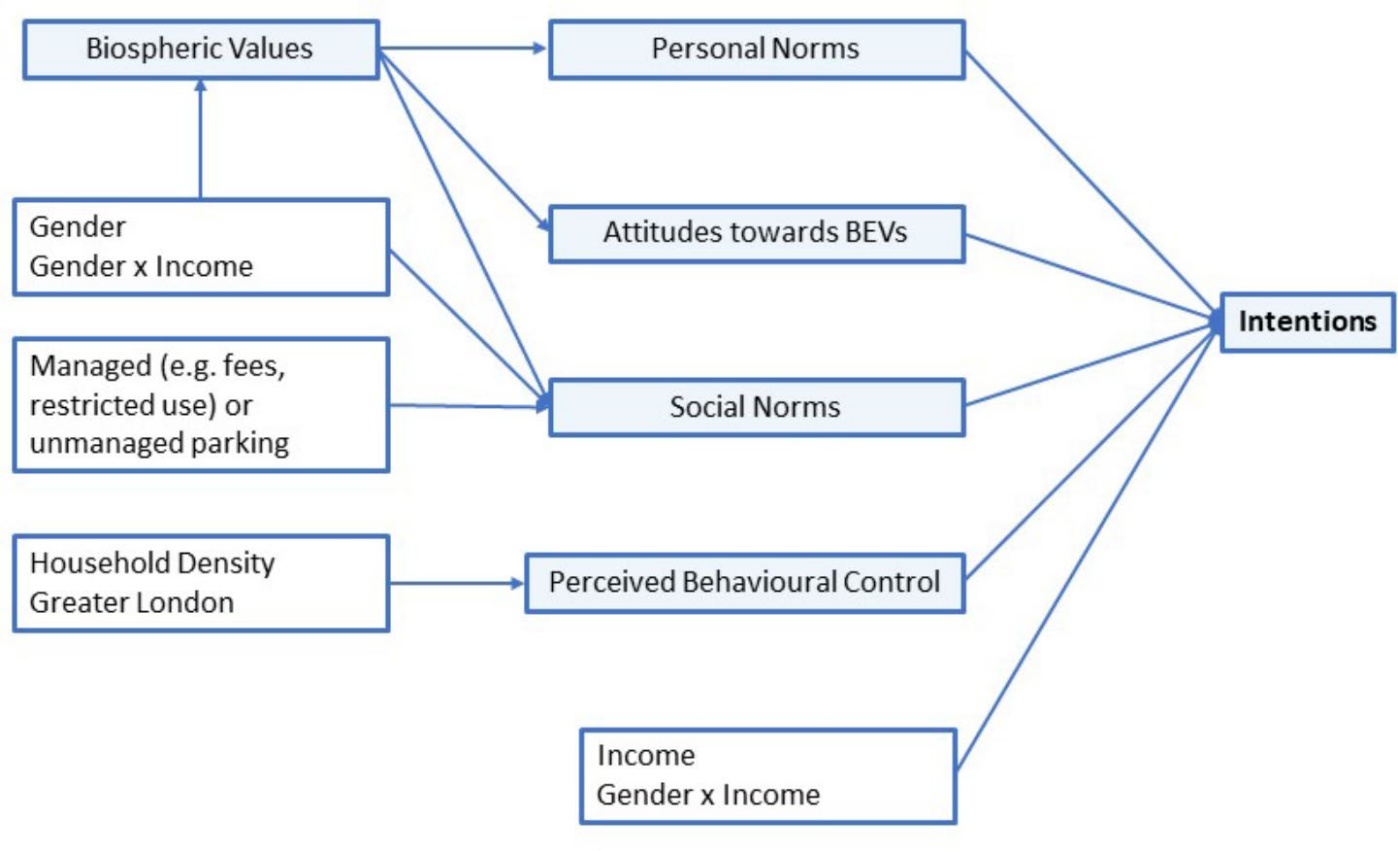
Final structural model of influences on intentions to adopt a BEV

The final structural model is shown in Fig. 3. The Satorra–Bentler scaled test statistic for this final model was χ^2^ = 731, *df* = 244, *p* < 0.00, with robust standard error estimations resulting in a root mean square error of approximation (RMSEA) of 0.041 with confidence intervals of 0.038–0.045, a standardised root mean square residual (SRMR) of 0.047, and a robust Tucker Lewis Index (TLI) of 0.956. Overall, this suggests a good fit of the model to the data.

The final SEM includes the direct influence of income, the ‘gender x income’ interaction term, norms, attitudes, and perceptions on intentions to adopt a BEV, and the indirect influence of biospheric values, gender, the ‘gender x income’ interaction term, unmanaged parking on-street, household density, and living in Greater London. The parking types described in Table 2 have been consolidated into a dummy variable that divides our sample into two fairly even halves of managed and unmanaged parking, with those who park on-street where there are no restrictions being assigned to the latter category and the rest to the former. The direct effects and calculated total effects are shown in Table 5, the latter in bold italics. We also include covariances between the psychological constructs in Table 6.

**Table 5:** Unstandardised coefficients in the structural model

↓From/To→		Biospheric Values	Personal Norm	Attitudes towards BEVs	Social Norm	Perceived Behavioural Control	Intent to adopt BEV
Biospheric values	D		0.576***	0.321***	0.150***		
	***T***		0.576***	0.321***	0.150***		0.441***
Personal Norm	D						0.503***
	***T***						0.503***
Attitudes towards BEVs	D						0.385***
	***T***						0.385***
Social Norm	D						0.187***
	***T***						0.187**
Perceived Behavioural Control	D						0.179***
	***T***						0.179***
Gender (Men coded as 1)	D	-0.113**			0.060**		
	***T***	-0.113**	-0.065**	-0.036*	***0.044***		***-0.038***
Income dummy (over £40k coded as 1)	D						− 0.014
	***T***						***-0.014***
Gender x Income interaction term	D	0.109			0.086**		0.159***
	***T***	***0.109***	***0.063***	***0.035***	0.102***		0.224***
Unmanaged parking dummy (On street, no restrictions coded as 1)	D				-0.112***		
	***T***				-0.112***		-0.021**
Household density	D					0.003**	
	***T***					0.003**	0.001*
Greater London dummy (live in GL coded as 1)	D					0.186***	
	***T***					0.186***	0.033**

Note: D = direct effect; T = total effect; *** *p* < 0.01; ** *p* < 0.05; *p < 0.10

**Table 6:** Unstandardised coefficients for covariances between psychological constructs

Covariances	Personal Norm	Attitudes towards BEVs	Social Norm
Attitudes towards BEVs	0.355***		
Social Norm	0.325***	0.163***	
Perceived Behavioural Control	0.094***	0.057***	0.069***

Note: *** *p* < 0.01; ** *p* < 0.05; *p < 0.10

As Table 5 shows, all psychological constructs in the extended TPB have a statistically significant effect on intention to adopt a BEV. However, effect sizes vary as standardised coefficients confirm: personal norm has the strongest effect (standardised coefficient [sc]: 0.527), followed by the total effect of biospheric values (sc: 0.385) and then attitudes (sc: 0.261). The effects of social norm and PBC are relatively small (sc: 0.103 and 0.077). Whilst our participants did indicate in their answers to the perceived behavioural control (PBC) items in Table 3 that they were not confident they would know how, where, and when to charge a BEV, our expectations that this would have a strong influence on intentions to adopt have not been fulfilled. Instead, the results suggest that biospheric values are driving feelings of personal obligation to adopt a BEV. Furthermore, the covariances in Table 6 show that personal norms, social norms, and attitudes are clearly interrelated (sc range between 0.540 and 0.724), whilst PBC is less related to any of the other three constructs (sc range between 0.236 and 0.338).

The importance of environmental motivations in shaping intentions to adopt a BEV is a finding common in the literature (Nayum et al. 2016; Rezvani et al. 2015; Schuitema et al. 2013; Simsekoglu and Nayum 2019). Our results build on this finding, showing that personal norms and attitudes that link BEV adoption to pro-environmental values and norms also have the greatest effect on conventional car drivers who are unlikely to be able to charge at home. By specifying PBC measures which focus on charging, rather than purchase, our results also show that the availability of public charging infrastructure has less influence on intentions than might have been expected, perhaps because of participants’ lack of knowledge about the practicalities of BEV ownership and use – including related to charging. A Norwegian study by Simsekoglu and Nayum (2019) suggested that the intentions of conventional car drivers to adopt BEVs are influenced by knowledge of the environmental and economic attributes of BEVs, whilst knowledge of the technical or practical aspects of BEVs (or lack thereof) had no statistically significant effects. Studies in Canada, Sweden and Denmark conclude that a lack of sensitivity to the availability, accessibility, and reliability of charging infrastructure could be due to a lack of awareness or familiarity with EVs and how public charging works (Haustein and Jensen 2018; Miele et al. 2020).

The effects of most of the sociodemographic and geographic variables on the different latent constructs and on intentions are small, but give an indication of how perceptions and norms related to BEV adoption are not homogeneous across the 1,281 individuals considered. Parking on-street without restrictions has a significant, negative effect on social norms (sc: -0.105), large enough to also have a significant, albeit small, negative effect on intentions (sc: -0.011, *p* = 0.01). Thus, parking in a managed space is positively associated with social norms, whether that parking was allocated or unallocated space in off-street, residential car parks and communal parking areas, or was on-street where there are restrictions, some of which involve payment. One possible explanation is that there is a greater awareness of BEVs and BEV charging in these contexts, perhaps due to increased social interactions generated by parking routines in such places (Kurnicki 2020). Alternatively, it might reflect social networks within wealthier neighbourhoods, where BEV adoption is higher and parking happens to be managed. An analysis of the spatial clustering of EV ownership in the UK showed that clusters correlated more with the socio-economic characteristics of neighbourhoods than with charging infrastructure density (Morton et al. 2018). These results show how the inequity of BEV adoption may be reinforced by the social norms that vary between wealthier and poorer neighbourhoods.

Meanwhile, the total effects of household density (sc: 0.007) and living in Greater London (sc: 0.011) on intentions are of a similar magnitude as that for the parking variable, but the effects are positive and mediated through PBC. Thus, those who live in areas of higher household density or those who live in Greater London are more confident that they would have control over charging their BEV compared to their counterparts in lower-density areas or outside Greater London. As Mukherjee and Ryan (2020) conclude in their Irish study, the relationship between urban living and EV adoption could be a reflection of neighbourhood characteristics. For example, the proactive installation and procurement of neighbourhood charging infrastructure is more common in Greater London and a selection of other proactive towns and cities facing residential parking pressures due to high density housing, among other factors. Furthermore, early competitive funding for charging infrastructure was awarded by the UK Government to certain London boroughs and a handful of other, mainly denser urban areas.^4^ On the other hand, the volume of public charging in Greater London may also be attributed to commercial providers, who are perhaps attracted to London’s status as a world city with high visibility. A virtuous cycle of EV adoption and private investment as found in German cities by Sommer and Vance (2021) could be observed in London at the time of our study, when it was still lacking in other parts of the UK, independent of household density.

Meanwhile, the ‘gender x income’ interaction term has direct and total (sc: 0.093) positive effects on intentions that are stronger than the effects for the other sociodemographic or geographic variables, even though the direct effect of income on intention and the total effect of gender on intention are both small and insignificant (even at *p* < 0.10). The conclusion is that higher income men have significantly stronger intentions to adopt a BEV than women or lower income men, and, as Table 5 shows, this is in large part due to differences in social norms. The effect of the interaction term on biospheric values is insignificant at *p* < 0.10, and the difference in this effect between women and higher income men is negligible. In comparison, the combined effects of gender and income on social norms are about twice as large as those on biospheric values. These stronger intentions which higher income men hold to adopt a BEV may be because BEV adoption is considered a status symbol among this group, a possibility also suggested by Haustein and Jensen (2018) in their study in Sweden and Denmark.

## Conclusions

Despite a plethora of studies on EV adoption, there is little consideration as to whether those who face the greatest constraints to charging a future BEV have different psychological motivations and barriers. Understanding this population is particularly relevant in European contexts like the UK if the policy goal of mass adoption is to be achieved. Hence, the main novelty of this paper concerns assessing the influence of residential parking in public or shared spaces on intentions to adopt BEVs. To do this, we extended previous socio-psychological studies of EV adoption (e.g. Simsekoglu and Nayum 2019) in the following ways:

- We targeted our survey at those who are unlikely to be able to easily charge a BEV from their domestic electricity and analysed the norms, attitudes and intentions of a nationally representative sample of car drivers without a private driveway or attached garage.
- We focused our construct of perceived behavioural control (PBC) on understandings of local EV charging opportunities, rather than including statements on the perceived ability to purchase a BEV.
- We included situational variables to reflect parking experience and public charging density, including the management of the usual, residential parking space; local household density; and a Greater London dummy.
- We considered the importance of analysing gender in conjunction with income when assessing intentions regarding BEVs.

Whilst Haustein and Jensen (2018) focus their PBC construct on charging-related statements, to the best of our knowledge, this is the first study based on a survey which specifically targets those with residential parking circumstances that make them unlikely to be able to charge at home. By also including contextual and parking-related variables in an extended TPB model and exploring their effects on different socio-psychological constructs, our methodological approach further accounts for the heterogeneity of built environments and how this might influence the spread of sustainability-related innovations like BEVs. Although recent studies have considered population density (Brückmann et al. 2021; Haidar and Rojas 2022), which had previously been highlighted as an area in need for further research, the only-parking related variable found in a review of over 200 articles on EV adoption is the presence of parking incentives for EV owners (Singh et al. 2020). Finally, while the effects of income and gender have often been considered in isolation in EV adoption studies (e.g. Chen et al. 2020; Wang et al. 2016), this study is unique in considering the interaction effects of gender and income. It shows that high income men hold stronger intentions to adopt a BEV than both low-income men and women with high or low incomes, and that this effect is primarily due to differences in social norms.

We conclude that among our sample focused on those who would likely be reliant on public charging, environmental motivations are paramount in shaping intentions to adopt a BEV (Klöckner 2014; Nayum et al. 2016; Wang et al. 2016), and that a lack of perceived behavioural control is less influential. The findings indicate this despite limited charging being a much-cited barrier to adoption (Biresselioglu et al. 2018; Singh et al. 2020; Wicki et al. 2023), particularly for groups without off-street parking at home. The policy implication of this finding is that for the target group of car drivers without a private driveway or garage, policies focused on reinforcing positive personal norms and attitudes are important to increase their adoption intentions. Messages emphasising how BEV adoption can help individuals contribute to climate action and cleaner air, and that BEVs are economical and fun to drive could bolster personal and social norms and positive attitudes towards BEVs. These could be targeted through events held in neighbourhoods where there is limited private parking or through second-hand BEV dealers, where the early and late majority of consumers may be more likely to purchase their vehicles. For example, a BEV presence at local green events^5^ alongside other examples of climate action can help conventional car drivers make the link.

The continued roll-out of public charging infrastructure for those who cannot charge at home is still needed to mitigate and ideally pre-empt concerns and anxiety over charging BEVs once an appetite for BEVs has been created among this group, but this should also be accompanied by better communication of the environmental and social benefits. Commitments to renewable energy use^6^ or the provision of public charging as a community asset could be better and more consistently publicised. Furthermore, as this study’s findings imply, charging infrastructure policy should take account not only of different parking circumstances and housing types, as recognised in the UK’s EV Infrastructure Strategy (DfT, 2022), but also of different neighbourhood characteristics, which have implications for more socially just policy interventions. Despite participants lacking a driveway or garage where they could park their car at home and potentially charge a future BEV, their norms and perceptions of BEVs varied according to their more specific residential parking and geographic situation. Living in Greater London or denser neighbourhoods affects charging expectations, which in turn influences intentions to adopt. On the other hand, social norms related to BEVs are weaker where drivers usually park on streets without restrictions. As White et al. (2022) describe in their study in American cities, the quantity and visibility of public charging infrastructure is more influential in terms of bolstering social norms related to EV adoption than in reducing range anxiety.

Therefore, it will be important to increase not only the availability and quantity of public charging outside London and in lower density areas with a lack of private parking, but also the visibility and accessibility of public charging infrastructure, particularly where unmanaged, on-street parking is common. This highlights the need for further research to inform such public charging infrastructure planning and management (Patil et al. 2023). A more in-depth study could unpick whether different residential parking management arrangements have different impacts on not only intentions to adopt BEVs, but also where adopters are likely to charge. Studying how widespread workplace parking and charging arrangements are for those who lack private home parking and charging opportunities would inform where alternatives are required mainly on weekends (Patil et al. 2023). Additional analysis into the relationship between paying for parking and paying for BEV charging would be useful, as those who already pay to park near home may perceive the addition of charging as less of a behavioural change. Further research could also explore the role of BEV experience at individual and neighbourhood levels, and how these impact attitudes and PBC (Haustein and Jensen 2018; White et al. 2022). Such investigations might uncover confounding spatial, temporal and social factors not included in our study.

Our study indicates norms, attitudes and perceptions among a group targeted by its residential parking at a single moment in time. This is a limitation of any cross-sectional study, but as the amount of available public charging infrastructure increases and BEV uptake accelerates, psychological motivations may also change rapidly. In addition to psychological motivations, it is important to consider that the price of BEVs can prevent intentions from becoming purchase decisions or environmental norms from translating into actual adoption (Corradi et al. 2023; Wicki et al. 2022). This relates to wider debates about low-income households – and women tend to be on lower incomes – trying to balance the affordability of car ownership with its necessity in car dependent places (Mullen, 2021). Although this study does control for income, further research is needed to investigate where low-income drivers live and how their mobility needs and experiences might be better addressed. Likewise, qualitative methods might be more appropriate to unpick the influence of not only gender, but also gender diversity and interactions with gendered roles within the household on values and norms related to BEV adoption. Consideration of these questions related to the equity of automobility should inform any assessment of whether financial incentives within BEV policy account for affordability obstacles faced by low income households or simply help pay for more luxury vehicles and the installation of personal charging infrastructure for their wealthy owners (Caulfield et al. 2022; Sovacool et al. 2019a).

In conclusion, this study set out to demonstrate that if the motivations of those most reliant on public charging infrastructure are not sufficiently understood, this could lead to (a) the exclusion of user groups with certain characteristics and parking circumstances and (b) the development of policy instruments that do not fit well with the normative expectations of these groups. Our findings suggest that the latter is occurring in the UK. Policy interventions are focused on increasing public charging infrastructure, without sufficiently considering how to foster norms related to BEV adoption and use of the local charging infrastructure among conventional car drivers who may be most reliant on it. The availability of public charging infrastructure to serve these drivers is important, but so are their environmental norms, values, and attitudes, which will shape their intentions to adopt a BEV. Only by stimulating awareness of the benefits and use of BEVs and encouraging the actualisation of pro-environmental motivations among groups with varying characteristics and parking circumstances, as well as addressing any perceived inability to charge locally, can BEV uptake be accelerated in an inclusive way.

## Electronic supplementary material

Below is the link to the electronic supplementary material.

Supplementary Material 1

## Footnotes

1 Many reviews, as well as some of the individual studies cited, refer to both BEVs and hybrid EVs or do not distinguish between them. Therefore, where the reference is to not solely BEVs, ‘EV’ is used as a more accurate abbreviation.

2 Household density was calculated using 2018 estimates from the Office for National Statistics and GIS-based measurements of the area of each postcode district.

3 Electric vehicles were described in the survey as those that “operate using a battery only, which must be recharged via an electrical outlet or charging station,” but abbreviated throughout the survey as ‘EV’ not ‘BEV’, as reflected in Fig. 2; Tables 3 and 4, where the exact wording of the survey is included.

4 The Go Ultra Low Cities scheme awarded £40 million to eight urban areas to implement measures supporting the adoption of EVs, including charging infrastructure: https://www.gov.uk/government/news/40-million-to-drive-green-car-revolution-across-uk-cities.

5 A programme for a local green event in Oxfordshire in 2021 that included EV and public charging information and test drives: https://eynsham.org.uk/variable/organisation/948/attachments/Eynsham’s-Great-Big-Green-Week-Detailed-Programme.pdf.

6 As in this Dutch campaign: Vandebron launches new EV campaign | MarketingTribune Agencies.
