# Supplementary material for: Electric vehicle adoption intentions among UK residents parking in shared and public spaces
Source: Transportation (Amst). 2024 Jul 29;53(2):937–60. doi: 10.1007/s11116-024-10518-0 (PMC12968124; doi:10.1007/s11116-024-10518-0)
Supplement: Supplementary file 1 — Supplementary Material 1 [file 11116_2024_10518_MOESM1_ESM.pdf]

Supplementary Information for article: Budnitz, H.*, Meelen, T., Schwanen, T. Electric vehicle adoption intentions among UK residents parking in shared or public spaces, *Transportation.*

*hannah.budnitz@ouce.ox.ac.uk; **Transport Studies Unit, University of Oxford**

Park and Charge Electric Vehicle Survey

Electric vehicles (EVs) are a promising technology that helps address problems such as air pollution. One of the main challenges surrounding the growth of EVs is the availability of infrastructure to charge them. Charging infrastructure may be hard to find for EV users who cannot park their car in a personal driveway or attached garage. As a possible solution, the Park and Charge project trials a service in which car parks close to residential areas are used for overnight charging of EVs by local residents.

[redacted] The University is conducting the current survey to understand your views on electric vehicles and how you would like to charge them if you had one. No background knowledge about electric vehicles is required. There are also questions about the car(s) you currently own and how you park your car(s) when at home.

This study has been reviewed by, and received ethics clearance through [redacted]

Please note that you may only participate in this survey if you are 18 years of age or over.

☐ I certify that I am 18 years of age or over

IF MOBILE DEVICE SHOW: **This survey is best undertaken on a tablet or a PC. If you do use a smartphone you can switch between desktop mode and mobile mode at any time by clicking the button at the bottom of the screen.**

We will first ask you some questions to check that you are eligible for the research. Any answer you give will be treated in confidence in accordance with the Code of Conduct of the Market Research Society.

Scoping Questions

1. Does your household currently own / lease one or more cars / vans? *A household is a group of people living together within one dwelling unit, whether house, apartment, or other form of accommodation even if unrelated.*
   ☐ Yes
   ☐ No THANK & CLOSE

2. Did you personally drive a car or van at least once in a typical week before the COVID-19 crisis?
   ☐ Yes
   ☐ No THANK & CLOSE

3. Do you have a driveway or attached garage at your home that fits ALL of the vehicles regularly driven by you or members of your household?
   ☐ Yes, for all our vehicles THANK & CLOSE
   ☐ No, not for all our vehicles

CHECK QUOTAS

4. What is your gender?

☐ Male
☐ Female
☐ Neither
☐ Other (please type in)

CHECK QUOTAS

Targets:	
Male	54%
Female	46%

4b What age group do you fall into?

☐ under 30 years old
☐ 30-50 years old
☐ Over 50 years old

CHECK QUOTAS

Targets:	
Under 30	20%
30-50	35%
50+	45%

5. Where do you live?

☐ East Midlands
☐ East of England
☐ Greater London
☐ North East
☐ North West
☐ Scotland
☐ South East
☐ South West
☐ Wales
☐ West Midlands
☐ Yorkshire and The Humber

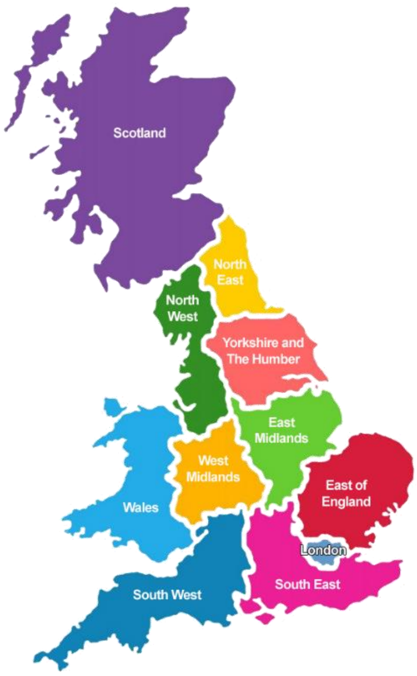

CHECK QUOTAS

QUOTA TARGETS	
East Midlands	8.0%
East of England	10.5%
Greater London	10.0%
North East	4.0%
North West	11.5%
Scotland	9.0%
South East	15.0%
South West	10.0%

Wales	5.0%
West Midlands	8.5%
Yorkshire and The Humber	8.5%

Thank you, I can confirm you are in scope for the survey.

The questionnaire will take about 30 minutes to complete and consists of five parts. You do not have to answer questions you do not wish to and you can terminate the questionnaire at any point. [Terms and conditions of participation / consent removed.]

If you have read the information above and agree to participate with the understanding that the data (including any personal data) you submit will be processed accordingly, please check the relevant box below to get started.

☐ Yes, I agree to take part

Thank you very much for agreeing to complete this on-line survey which is conducted by [redacted]

For convenience you can stop and return to complete the questionnaire as many times as you wish, although once submitted you will not be able to enter again.

Part I: Parking

Please answer the following questions about your daily parking.

6. How many adults are there in your household who drive?
   1
   2
   3
   4
   5+

7. How many cars / vans does your household own / lease?

   ______ cars
   ______ vans

8. How would you describe your home?
   ☐ Detached
   ☐ Semi-detached
   ☐ Terrace
   ☐ Flat
   ☐ Other (please type in)

9. What is the tenure of your home?
   ☐ Owner-occupied
   ☐ Privately rented
   ☐ Socially rented
   ☐ Shared ownership
   ☐ Other (please type in)

Now consider the vehicle you drive and park most often.

10. Where do you usually park your vehicle near your home?
    ☐ private garage, driveway
    ☐ Personal space in a car park
    ☐ Car park or parking area without allocated space
    ☐ On street in a controlled parking zone (e.g. residents' parking zone)
    ☐ On street, where there are no restrictions
    ☐ Other (please type in)

11. How close to your home is the location where you usually park?
    ☐ Directly outside my home
    ☐ Less than 2 minutes' walk to my home
    ☐ Between 2 and 5 minutes' walk
    ☐ Between 5 and 10 minutes' walk
    ☐ Over 10 minutes' walk
    ☐ Varies too much to pick one of the above

12. How long did you usually park your vehicle near your home on *weekdays* prior to the COVID-19 crisis?
    ☐ Overnight only, from the evening of the previous day until the next morning
    ☐ Overnight as well as for periods of a few hours at a time during the day
    ☐ Multiple days (and nights) in a row
    ☐ Varies too much to pick one of the above
    ☐ Don't Know

13. Do you pay for parking near your home (e.g. for a residents' permit or to rent a garage or space in a car park)?
    ☐ Yes
    ☐ No **GO TO Q15**
    ☐ Don't Know **GO TO Q15**

14. How much do you pay per year?
    ☐ Less than £50
    ☐ £50-£100
    ☐ £100-£500
    ☐ Over £500
    ☐ Don't know

Now we would like to ask you about your parking at home. Please tell us to what extent you agree or disagree with the following statements. *RANDOMISE ORDER*

15. About parking space in your neighbourhood or the area where you live:

a. There is enough parking space available in my neighbourhood for everyone who needs it.
   Strongly disagree   Disagree   Neither agree nor disagree   Agree   Strongly agree
b. Parked vehicles make it difficult to walk around my neighbourhood.
   Strongly disagree   Disagree   Neither agree nor disagree   Agree   Strongly agree
c. I feel safe parking my vehicle in my neighbourhood.
   Strongly disagree   Disagree   Neither agree nor disagree   Agree   Strongly agree
d. I often feel as if I'm competing with my neighbours for a parking space.
   Strongly disagree   Disagree   Neither agree nor disagree   Agree   Strongly agree
e. My household chooses to have fewer vehicles because there is limited parking near my home.
   Strongly disagree   Disagree   Neither agree nor disagree   Agree   Strongly agree

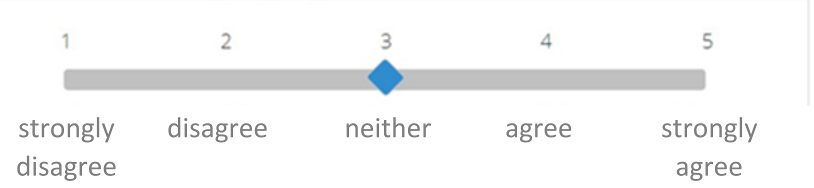

16. **About parking in your neighbourhood:** *RANDOMISE ORDER*

a. I often feel stressed when searching for a parking space near my home.
   Strongly disagree   Disagree   Neither agree nor disagree   Agree   Strongly agree
b. It is easy for me to walk home after parking my vehicle.
   Strongly disagree   Disagree   Neither agree nor disagree   Agree   Strongly agree
c. I drive a smaller vehicle than I'd like because of limited parking space near our home.
   Strongly disagree   Disagree   Neither agree nor disagree   Agree   Strongly agree
d. I get anxious when I can only find a narrow parking space or when I have to parallel park.
   Strongly disagree   Disagree   Neither agree nor disagree   Agree   Strongly agree

e. I am confident I know where to find a parking space in my neighbourhood when I need it.

Strongly disagree Disagree Neither agree nor disagree Agree Strongly agree

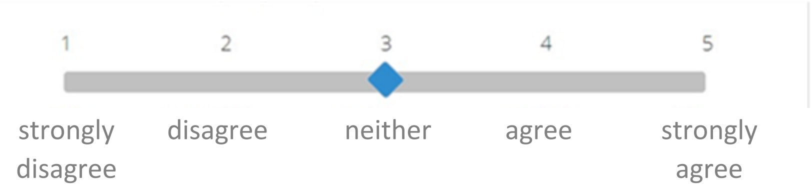

17. **About parking in different places:** *RANDOMISE ORDER*

1. I would park in a car park instead of on-street near my home if I knew empty spaces were always available.

   Strongly disagree Disagree Neither agree nor disagree Agree Strongly agree

2. I would be willing to walk further to park my vehicle if I knew it were in a safer place (e.g. because it was monitored by CCTV).

   Strongly disagree Disagree Neither agree nor disagree Agree Strongly agree

3. Buying a new vehicle would make me consider changing where I usually park when at home.

   Strongly disagree Disagree Neither agree nor disagree Agree Strongly agree

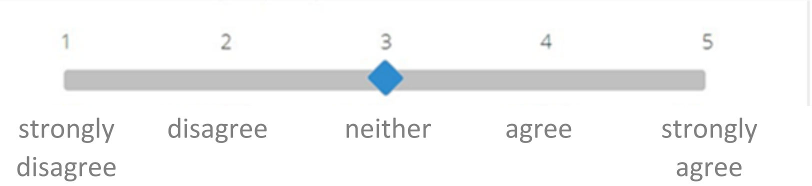

Part II: Electric Vehicles

The next section of the questionnaire asks if you have any experience of electric vehicles and charging them. Pure electric vehicles operate using a battery only, which must be recharged via an electrical outlet or charging station. Outlets can be the same type as those used for other electrical appliances and devices, but the speed of recharging a vehicle is slow. The speed available at public charging stations varies, but is usually faster than charging at home.

[graphic redacted]

Please indicate any experience you have with an electric vehicle (EV).
Tick all that apply.

- ☐ I currently own / lease an EV
- ☐ I used to own / lease an EV, but no longer do
- ☐ I have driven an EV
- ☐ I have been a passenger in an EV
- ☐ I know someone personally who owns an EV
- ☐ I have seen EVs in my neighbourhood
- ☐ I am interested in EVs and have looked up information on them
- ☐ I have seen EVs in the news or in advertisements
- ☐ None of the above

18. When do you expect to purchase / lease your next vehicle?
    - ☐ Within the next year
    - ☐ Between 1 and 5 years from now
    - ☐ Over 5 years from now
    - ☐ Not sure / Don't know
    - ☐ Don't expect to purchase / lease another vehicle

19. About your intentions to switch to an electric vehicle (EV). *Please indicate to what extent you agree or disagree with the following statements.* RANDOMISE ORDER

a. I plan to replace my current vehicle with an EV.
Strongly disagree Disagree Neither agree nor disagree Agree Strongly agree

b. I plan to reduce the number of vehicles my household has and join a car club with EVs.
Strongly disagree Disagree Neither agree nor disagree Agree Strongly agree

c. I will seriously consider leasing or buying an EV when I need a new vehicle.
Strongly disagree Disagree Neither agree nor disagree Agree Strongly agree

d. For my next vehicle, I want to buy / lease an EV.
Strongly disagree Disagree Neither agree nor disagree Agree Strongly agree

e. I intend to join a car club which allows me to drive EVs as soon as one is locally available.
Strongly disagree Disagree Neither agree nor disagree Agree Strongly agree

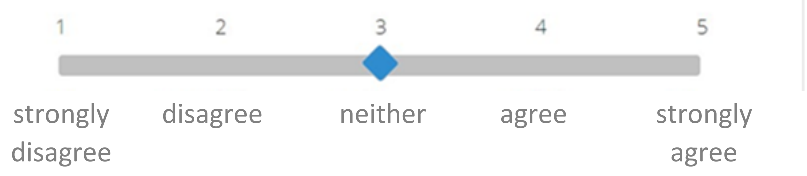

20. Please tick all the types of places where you have seen charge points or stations for EVs:
    ☐ At my home
    ☐ At the homes of friends, family or neighbours
    ☐ At workplaces
    ☐ At supermarkets
    ☐ At other retail locations / destinations (e.g. gyms, churches, cinemas, etc)
    ☐ At refuelling stations / motorway service stations
    ☐ Public / Council car parks
    ☐ On street
    ☐ Other (please type in)
    ☐ I have never seen an EV charging station or point.

21. Even if you do not have any experience with EVs, you may have formed views about them. Please indicate to what extent you agree or disagree with the following statements *RANDOMISE ORDER*

a. EVs are too expensive to purchase.
   Strongly disagree Disagree Neither agree nor disagree Agree Strongly agree
b. EVs are a good option because they are cheap to run and maintain.
   Strongly disagree Disagree Neither agree nor disagree Agree Strongly agree
c. I would feel a strong personal obligation to buy / lease an EV for my next vehicle to reduce carbon emissions and improve air quality.
   Strongly disagree Disagree Neither agree nor disagree Agree Strongly agree
d. EVs are an exciting new technology.
   Strongly disagree Disagree Neither agree nor disagree Agree Strongly agree
e. When driving an EV, I would always be worried about running out of charge.
   Strongly disagree Disagree Neither agree nor disagree Agree Strongly agree
f. Many of the people who are important to me (friends, family) own fuel efficient and environmentally friendly vehicles.
   Strongly disagree Disagree Neither agree nor disagree Agree Strongly agree
g. Over the long term, an EV is a cheaper option than a diesel or petrol vehicle.
   Strongly disagree Disagree Neither agree nor disagree Agree Strongly agree
h. I would feel guilty if I did not purchase an EV for my next vehicle to reduce carbon emissions and improve air quality.
   Strongly disagree Disagree Neither agree nor disagree Agree Strongly agree
i. EVs do not come in the size or shape of vehicle that suit my needs (e.g. too small for family).
   Strongly disagree Disagree Neither agree nor disagree Agree Strongly agree
j. I think it would be difficult to park and charge an EV near my home if my household bought / leased one.
   Strongly disagree Disagree Neither agree nor disagree Agree Strongly agree
k. People whose opinions I value would prefer that I adopt an EV when adopting a vehicle in the near future
   Strongly disagree Disagree Neither agree nor disagree Agree Strongly agree
l. I like the sound and power of a conventional vehicle engine.
   Strongly disagree Disagree Neither agree nor disagree Agree Strongly agree
m. I wouldn’t know how to charge an EV near my home.
   Strongly disagree Disagree Neither agree nor disagree Agree Strongly agree
n. EVs are more comfortable to drive than a conventional vehicle.
   Strongly disagree Disagree Neither agree nor disagree Agree Strongly agree
o. Because of my own principles, I feel I should choose an EV for my next vehicle to reduce carbon emissions and improve air quality.

Strongly disagree Disagree Neither agree nor disagree Agree Strongly agree

p. People who are important to me expect me to choose an EV for my next vehicle.

Strongly disagree Disagree Neither agree nor disagree Agree Strongly agree

q. In my neighbourhood, I don't know where I could charge an EV.

Strongly disagree Disagree Neither agree nor disagree Agree Strongly agree

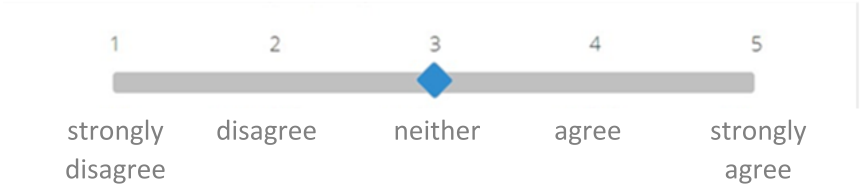

Let's now imagine that you have decided to purchase an EV. Now you need to think about where you will charge it when it is low on battery.

22. Where do you think you would be willing and able to charge your EV? *Think about what is currently available, convenient, or is likely to be locally installed in the near future. Please tick all that apply:*

☐ At my home
☐ At the homes of friends, family or neighbours
☐ At workplaces
☐ At supermarkets
☐ At other retail locations / destinations (e.g. gyms, churches, cinemas, etc)
☐ At refuelling stations / motorway service stations
☐ Public / Council car parks
☐ On street
☐ Other (please type in)

Part III: Charging Choices

Let's continue to imagine that you have decided to get an electric vehicle (EV).

We will now explore the trade-offs that you might have to make when deciding where to charge your new EV. We will ask you to choose between different options for parking and charging your EV at home over night. Assume that if you charge overnight, a full charge is guaranteed.

If an EV owner cannot charge from their home electricity, various cities around the UK are now offering on-street charging from bollards or lampposts.

[photo redacted]

These may be accompanied by designated bays and are operated by various companies. One option in each choice you are given below will assume that these sorts of facilities are available in your neighbourhood and you can choose to charge on-street near your home.

The other option in each choice is a charging station installed in a car park near your house that is well-used during the day, but often empty at night.

[photo redacted]

The options you are provided differ in terms of the following:

- whether you can be certain that a charging space is available for you whenever you want it;
- the cost of each overnight charging session, including both the space and the electricity for charging the EV's battery back to its full range;
- how you can pay for these charging sessions;
- how long your EV is allowed to remain parked in the charging space when not charging;
- the security provisions for your EV whilst charging;
- the average time it would take you to walk from the charging space to your home; and
- how you might experience your walk home from the charging space.

The aim of this exercise is to encourage you to consider your preferences carefully and decide which of the two options is best for you in each choice you are given. You may like some parts more and other parts less, but you still need to decide which option you would prefer overall.

**Attribute**	**On-Street**	**Car Park**
Charging space guarantee	• No guarantee<br>•<br>•	• No guarantee<br>• Space reserved on booking<br>• Unique space only allocated to you
Charging fee (per night, to full battery)	• £4<br>• £8<br>• £12	• £4<br>• £8<br>•
Payment	• Per charging session (pay as you go)<br>•	• Per charging session (pay as you go)<br>• Monthly (subscription)
Parking duration	• Unlimited<br>•<br>• 8pm-8am only	•<br>• 5pm-9am only<br>• 8pm-8am only
Dedicated security measures at parking location	• None<br>• CCTV<br>•	• None<br>• CCTV<br>• Security guards on patrol
Walk time to home	• 2 minutes<br>• 5 minutes<br>• 10 minutes	• 2 minutes<br>• 5 minutes<br>• 10 minutes
Walk experience	• Good experience: the route is pleasant and you feel safe<br>• Neutral: route is OK and makes you neither safe nor unsafe<br>• Bad experience: the route is unpleasant and makes you feel anxious	• Good experience: the route is pleasant and you feel safe<br>• Neutral: route is OK and makes you neither safe nor unsafe<br>• Bad experience: the route is unpleasant and makes you feel anxious

Part IV: Your Vehicle Use and Other Transport Services

Now please answer a few more questions about your current vehicle use.

1. Approximately how many miles would you usually drive per day, prior to the COVID-19 crisis?

   miles

   Don't know

2. How often did you drive more than 30 miles in one day, prior to the COVID-19 crisis?
   ☐ Less than once a year to never
   ☐ Less than once a month to once a year
   ☐ Less than once a week to once a month
   ☐ Once or twice a week
   ☐ Three or more times a week
   ☐ Don't know

3. Over the last year, what is the longest trip you took by private vehicle in one day? *If you came back the same day, please include both directions. Please enter your best estimate as a whole number, in miles.*

   miles

   ☐ Don't Know

4. How much did you normally spend on fuel per month, prior to the COVID-19 crisis?
   ☐ Less than £50
   ☐ £50-£100
   ☐ over £100
   ☐ Don't Know

5. How often did you normally refuel your vehicle in the same location (petrol station, garage), prior to the COVID-19 crisis?
   ☐ Almost always at the same location
   ☐ Almost always in the same 2-3 locations
   ☐ Rarely in the same location
   ☐ Don't know

6. When do you usually refuel your vehicle?
   ☐ When there is a third of a tank or more fuel left
   ☐ When there is between a quarter and a third of a tank left
   ☐ When there is less than a third of a tank left
   ☐ When near my preferred refuelling station
   ☐ Other_______________

EVs are not the only growing technology trend in transport. Please tell us about your experiences with delivery and transport apps and shared transport services.

7. In the last year, how often did you use an app to book and pay for a service such as train tickets, taxi booking, home delivery, prior to the COVID-19 crisis?

☐ Three or more times a week
☐ Once or twice a week
☐ Less than once a week to once a month
☐ Less than once a month to once a year
☐ Less than once a year to never

8. In the last year, how often did you use a shared transport service such as a car club vehicle or a bike hire, prior to the COVID-19 crisis?
☐ Three or more times a week
☐ Once or twice a week
☐ Less than once a week to once a month
☐ Less than once a month to once a year
☐ Less than once a year to never

Part V: About You

In this section, we are asking for further information about you as who you are and where you live affects parking circumstances and the potential for mass adoption of EVs. All personal information will remain confidential and reporting of results will be aggregated, anonymised, and otherwise not disclose any personal information. We also offer a 'prefer not to answer' option for personal questions, which will enable you to proceed to the next question.

9. What is the first part of your postcode of your home? *For example, if your postcode is GU20 6PF please enter GU 20 6 below.*

We are asking for this information in order to understand more about the geography of where people are unable to charge an EV at home and need alternative EV charging solutions.

example	GU	20		6
	**DROP DOWN**			

Q9b Can you please provide the last part of your home postcode in the following two boxes.

**DROP DOWN**	**DROP DOWN**

☐ I prefer not to answer.

1st DROP DOWN BOX

AB	BT	DA	EH	HP	KY
AL	CA	DD	EN	HR	L
B	CB	DE	EX	HS	LA
BA	CF	DG	FK	HU	LD
BB	CH	DH	FY	HX	LE
BD	CM	DL	G	IG	LL
BH	CO	DN	GL	IP	LN
BL	CR	DT	GU	IV	LS
BN	CT	DY	HA	KA	LU
BR	CV	E	HD	KT	M
BS	CW	EC	HG	KW	ME

MK	OX	S	SR	TR	WR
ML	PA	SA	SS	TS	WS
N	PE	SE	ST	TW	WV
NE	PH	SG	SW	UB	YO
NG	PL	SK	SY	W	ZE
NN	PO	SL	TA	WA	
NP	PR	SM	TD	WC	
NR	RG	SN	TF	WD	
NW	RH	SO	TN	WF	
OL	RM	SP	TQ	WN	

10. What is your year of birth? ________________*DROP DOWN LIST FROM 1915-2002*
☐ I prefer not to answer.

11. What is your highest education level?
☐ Degree or Higher Degree level qualifications (e.g. BA, MSc, PhD), HND, NVQ4 (or equivalent)
☐ NVQ3, 2 or more A levels, advanced GNVQ, Scottish higher (or equivalent)
☐ NVQ2, 5 or more GCSEs at grades A-C, intermediate GNVQ, Scottish intermediate 2 (or equivalent)
☐ NVQ1, fewer than 5 GCSEs at grades A-C, foundation GNVQ, Scottish intermediate 1 (or equivalent)
☐ Other qualifications______________________________
☐ No formal qualifications

12. Which of these best described your work situation prior to the COVID-19 crisis?
☐ Doing paid work full-time as an employee (35 or more hours per week)
☐ Doing paid work part-time as an employee (less than 35 hours per week)
☐ Self-employed or freelance
☐ On temporary leave (e.g. due to maternity, illness)
☐ Unemployed
☐ Full-time student
☐ Retired
☐ Looking after home or family
☐ Long term sick or disabled
☐ Other (please specify) ____________

13. Last year, what was your annual household income from all sources (including salaries, pensions, benefits, etc) before tax? ⓘ
ⓘ We ask for this information because EV drivers tend to be wealthier and have access to home charging. Part of the purpose of this project is to test the potential for all income groups to be part of the transition to electric mobility and have good and affordable EV charging options. We want to understand how different income groups may view EVs and the park and charge choices we have proposed.
☐ Up to £20,000
☐ £20,001 - £40,000
☐ £40,001 - £60,000
☐ £60,001 - £80,000
☐ Over £80,000
☐ Prefer not to say

14. How many people live in your household, including yourself?
Children aged under 16 (Drop down menu: 0 (default) 1 2 3 4 >4)

Adults aged 16 and over, including yourself (Drop down menu: 1 2 3 4 >4)

Finally, consider the statements below and indicate how important each is as a guiding principle in your life.

Wealth: material possessions, money

(Not important at all) 1….2….3….4….5 (Very important)

Authority: the right to lead or command

(Not important at all) 1….2….3….4….5 (Very important)

Equality: equal opportunity for all

(Not important at all) 1….2….3….4….5 (Very important)

Social justice: correcting injustice, care for the weak

(Not important at all) 1….2….3….4….5 (Very important)

Preventing pollution: protecting natural resources

(Not important at all) 1….2….3….4….5 (Very important)

Protecting the environment: preserving nature

(Not important at all) 1….2….3….4….5 (Very important)

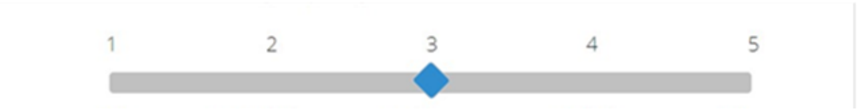

Thank you for your time.
